# Fumigation of Brazil nuts with allyl isothiocyanate to inhibit the growth of Aspergillus parasiticus and aflatoxin production

**DOI:** 10.1002/jsfa.8527

**Published:** 2017-08-12

**Authors:** Lucas F Lopes, Keliani Bordin, Gabriel HC de Lara, Federica Saladino, Juan M Quiles, Giuseppe Meca, Fernando B Luciano

**Affiliations:** ^1^ School of Life Sciences Pontifícia Universidade Católica do Paraná Curitiba, Paraná Brazil; ^2^ Laboratory of Food Chemistry and Toxicology, Faculty of Pharmacy University of Valencia Burjassot Spain

**Keywords:** natural products with biocidal activity, food quality, food safety, mycotoxins, shelf life

## Abstract

**BACKGROUND:**

Brazil produces approximately 40 000 tons of Brazil nuts annually, which is commonly contaminated with fungi and mycotoxins. Gaseous allyl isothiocyanate (AITC) was used to inhibit the growth of Aspergillus parasiticus and its production of aflatoxins (AFs) in Brazil nuts.

**RESULTS:**

Nuts were inoculated with 10^4^ spores g^−1^ of A. parasiticus and placed in airtight glass jars with controlled relative humidity (RH = 95 or 85%). Samples were treated with 0, 0.5, 1.0 or 2.5 µL L^−1^ of gaseous AITC and analyzed after 30 days to determine the fungal population and AFs content. Samples were also submitted to sensory evaluation. AITC at 2.5 µL L^−1^ could completely inhibit the fungal growth and AFs production in both the RH tested. AITC at 0.5 and 1 µL L^−1^ did not affect the microbial growth at RH = 95%, but 1 µL L^−1^ reduced the production of AFs by ∼50%. All AITC treatments reduced the fungal population and AFs to undetectable levels at RH = 85%. None of the concentrations altered sensory characteristics of Brazil nuts.

**CONCLUSION:**

Gaseous AITC could be used as an alternative to inhibit the growth of A. parasiticus during storage and transport of Brazil nuts. © 2017 The Authors. *Journal of the Science of Food and Agriculture* published by John Wiley & Sons Ltd on behalf of Society of Chemical Industry.

## INTRODUCTION

Brazil nuts are well known for their high content of selenium, proteins, vitamins and unsaturated fatty acids.^1^ They are the seeds of *Bertholletia excelsa*, which is commonly found in the Amazon region, especially in the Brazilian state of Acre. Flowering occurs during the dry season, between August and October, and harvesting is done during the wet season, between November and March.^2^ The production area of this seed in the Amazon region comprises 325 million hectares, generating 40 643 tons of Brazil nuts in 2015 with a production value of US$ 35.8 million.^3^ Approximately 40% of this production is exported and the main importers are the United States, Germany and Spain.^4^, ^5^

Harvest of Brazil nuts is performed mainly by under‐privileged families, using little technology, which results in a high incidence of microbial contamination.^6^ The high humidity and temperatures of the Amazon region in combination with the transport of the nuts in precarious trucks set up ideal conditions for fungal development. Moreover, the presence of toxigenic *Aspergillus* in Brazil nuts is common, especially *Aspergillus parasiticus* and *Aspergillus flavus*.^7^ Recent restrictive measures of the European Community regarding the permissible limits of aflatoxins (AFs) in Brazil nuts resulted in >90% reduction in the exportation of this product.^8^

*A. parasiticus* is a filamentous fungus which produces AFs B_1_, B_2_, G_1_ and G_2_, which are secondary metabolites with carcinogenic activity.^2^ The maximum tolerated level for the presence of total AFs in raw Brazil nuts for direct consumption is 10 µg kg^−1^ in Brazil, while in the European market this limit is 4 µg kg^−1^ for total AFs and 2 µg kg^−1^ for AFB_1_.^8^ Food preservatives and modified atmosphere packaging are commonly used to avoid the growth of filamentous fungi in nuts. More recently, natural alternatives have been sought to substitute synthetic antimicrobials in foods. Allyl isothiocyanate (AITC) is a natural compound from mustard, horseradish, wasabi and many cruciferous vegetables. AITC presents potent antimicrobial activity towards bacteria and fungi in various food matrices,^9^, ^10^, ^11^, ^12^, ^13^ including nuts.^14^, ^15^ This compound has a ‘Generally Recognized as Safe’ status for human consumption by the US Food and Drug Administration.^16^ Moreover, AITC is a volatile compound that can be used in the gaseous form to inhibit the growth of filamentous fungi. Saladino *et al*.^17^ used gaseous AITC formed directly from oriental mustard flour to avoid the growth of *Aspergillus parasiticus* in Italian piadina and the gas was able to reduce the production of AFB_1_, AFB_2_, AFG_1_ and AFG_2_ by >70%. AITC was also used to fumigate peanuts contaminated with *A. parasiticus* and AFs production was also avoided.^14^ The objective of the present study was to apply gaseous AITC in Brazil nuts in order to inhibit the growth of *Aspergillus parasiticus* as well as the production of aflatoxins at doses that do not affect the sensory characteristics of the product.

## MATERIALS AND METHODS

### Chemicals and microbiology material

#### Chemicals

AFs B_1_, B_2_, G_1_ and G_2_, AITC (95%), acetonitrile, methanol, potassium nitrate, potassium chloride, sodium chloride and tartaric acid were purchased from Sigma–Aldrich (St. Louis, MO, USA) and were all HPLC‐grade. Deionized water (<18 MΩ cm resistivity) was obtained from a Milli‐Q water purification system (Millipore, Bedford, MA, USA). Chromatographic solvents and water were degassed for 15 min at 20 °C using a Branson 5200 (Branson Ultrasonic Corp., Danbury, CT, USA) ultrasonic bath. *A. parasiticus* CECT 2681 was obtained from the Spanish Type Culture Collection (CECT, Valencia, Spain). Buffered peptone water, potato dextrose agar (PDA) and potato dextrose broth (PDB) were provided by Liofilchem (Roseto degli Abruzzi, Italy).

#### Material

A mirrored Neubauer chamber (Blaubrand 7186 05; Wertheim, Germany) was used for fungal spore counting. Ultraturrax IKA T18 (IKA Works, Staufen, Germany); evaporators R‐200 from Büchi Rotavapor (Postfach, Switzerland) and Turbovap LV Evaporator from Zymark (Hopkinton, MA, USA) were used for mycotoxin extraction. Jars used for fumigation of Brazil nuts were obtained from Analisis Vinicos (Tomelloso, Spain).

### Preparation of Aspergillus parasiticus inoculum

*A. parasiticus* CECT 2681 was maintained at −80 °C before use. Then, it was thawed at room temperature, suspended in 10 mL of PDB and incubated for 48 h at 25 °C. Aliquots of 200 µL of this culture were inoculated in a Petri dishes containing PDA, which were incubated for 7 days at 25 °C for fungal growth and sporulation. Petri dishes were added with 5 mL of sterile 9 g L^−1^ NaCl solution and stirred to collect the largest number of spores possible. Then, the supernatant was transferred to 15 mL falcon tubes and 100 µL of this solution was transferred to a mirrored Neubauer chamber (Blaubrand 7186 05; Wertheim, Germany) for spore counting using an optical microscope. Spore numbers were diluted accordingly in sterile saline solution to produce the inoculation solution.

### Packaging and treatment of Brazil nuts with AITC

**Figure 1:**
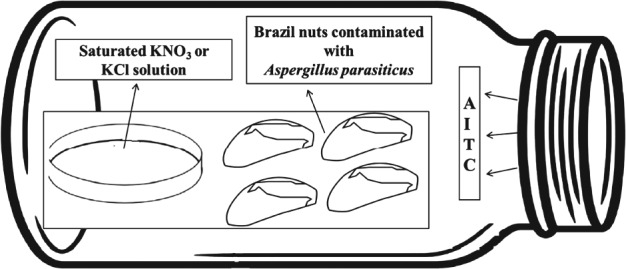
Scheme of the fumigation process. Brazil nuts contaminated with Aspergillus parasiticus and Petri dish lid containing KNO_3_ or KCl solution were placed on top of a rigid base support. Filter papers containing different concentrations of AITC were adhered in the inner part of the jar lids. The jar was hermetically closed and volatilization of AITC occurred at room temperature.

Brazil nuts were portioned in samples of 25 g that were contaminated with *A. parasiticus* at a final concentration of 10^4^ spores g^−1^. These samples were individually placed on top of a rigid base support, which were transferred to 1‐L glass jars (Analisis Vinicos, Spain). In addition, 50‐mm Petri dish lids containing 15 mL of saturated KNO_3_ or KCl solution was also placed on the rigid base support to generate a RH of approximately 95% or 85% inside the jar, respectively, simulating the tropical environment of Amazon region, where nuts are harvested and transported (Fig. 1). Finally, filter papers of 2.5 × 2.5 cm containing different concentrations of AITC 95% were prepared and adhered in the inner part of the jar lids. The volume of AITC added to the filter papers was calculated to generate 0.5, 1 or 2.5 µL L^−1^ after total volatilization. Control samples were prepared without AITC. Then, jars were hermetically closed to allow the fumigation of the nuts. Experiments were performed three times in triplicates for both RH tested. Samples were kept under observation for 30 days and the first sign of visual fungal growth was considered the end of the shelf life of the product.

### Microbiological analysis

After 30 days of observation, 20 g of each sample were combined with 180 mL of 0.9% NaCl sterile solution in a stomacher bag (VWR International BVBA, Leuven, Belgium) and homogenized for 1 min (Stomacher IUL Instrument; Barcelona, Spain). Serial dilutions were prepared and 100 µL of each dilution was inoculated in duplicates in PDA acidified to pH 3.5 using a 100 g L^−1^ tartaric acid solution. Petri dishes were incubated at 25 °C for up to 7 days before colony counting according to the Brazilian legislation.^18^

### Aflatoxins extraction

Aflatoxins extraction was performed using the method described by Hontanaya *et al*.^14^ with few modifications. Briefly, 5 g of finely ground nuts (Oster Classic grinder; Oster, Valencia, Spain) were weighed in a 50 mL plastic tube. Then, 25 mL of methanol was added. The samples were extracted using an Ultra IKA T18 basic Ultraturrax (IKA Works) for 3 min. The mixture was centrifuged at 1700 × *g* for 5 min and the supernatant was evaporated to dryness at 40 °C with a Büchi Rotavapor R‐200. The residue was dissolved with 5 mL of methanol, transferred to a 15 mL plastic tube and evaporated to dryness using a multi‐sample nitrogen evaporator Turbovap LV Evaporator (Zymark) operating at 35 °C and 5 psi nitrogen pressure. After solvent evaporation, the extract was reconstituted with 1 mL of methanol, filtered through a 13 mm/0.22 µm filter and injected into high‐performance liquid chromatography with fluorescence detection (HPLC‐FLD).

### HPLC‐FLD analysis

AFs were determined using HPLC (Merck, Darmstadt, Germany) equipped with a fluorescence light detector RF‐10Axl (Shimadzu, Kyoto, Japan) and Hitachi Software Model D‐7000 version 4.0 was used for data analysis. A Gemini C18 column (Phenomenex, Torrance, CA, USA) 4.6 × 150 mm, 3 µm particle size was used as the stationary phase. The isocratic mobile phase was consisted of water/methanol/acetonitrile (60:20:20, v/v/v) with a flow rate of 0.5 mL min^−1^. The fluorimeter detector was set up at *λ*_ex_ of 366 nm and *λ*_em_ of 428 nm. Calibration curves were determined by injecting each mycotoxin at different concentrations (1 µg L^−1^ to 10 000 µg L^−1^), relating the area of the peak with the quantity of mycotoxin. A filtered matrix of Brazil nuts was spiked with known concentrations of AFB_1_, AFB_2_ and AFG_2_ standards to avoid possible errors in the retention times of aflatoxins caused by the matrix effect. Then, each sample was analyzed in the HPLC‐FLD system for identification and quantification of AFs. Validation method parameters are presented in Table 1.

**Table 1:** Results of validation method parameters for detection and quantification of AFB_1_, AFB_2_, AFG_1_, AFG_2_ in Brazil nuts

Aflatoxin	Spiked (µg kg^−1^)	Recovery	ME (%)	LOD	LOQ	Repeatability (RSD %)	Reproducibility (RSD %)
B_1_	0.5	90.3	88.2	0.05	0.5	86.4	82.3
	2.0	94.5	90.4			88.3	81.1
	5	96.8	94.2			89.3	84.2
B_2_	0.5	88.2	83.4			82.4	80.3
	2.0	89.3	85.9			84.1	77.3
	5	90.4	88.6			83.4	75.2
G_1_	0.5	93.1	89.0			88.5	82.0
	2.0	95.8	90.4			87.9	83.4
	5	93.0	93.4			85.0	82.4
G_1_	0.5	91.2	92.1			89.3	80.3
	2.0	93.2	93.4			90.2	81.3
	5	90.2	95.3			91.2	84.6

Spiked, concentration spiked in Brazil nuts; ME, matrix effect; LOD, limit of detection; LOQ, limit of quantification.

### Determination of moisture content in Brazil nuts

Moisture content of Brazil nuts stored at 85% and 95% of RH was determined after 0, 1, 2, 3, 7, 10, 16, 20, 25 and 30 days. Samples used for this test were not inoculated with *A. parasiticus*, but were submitted to the same conditions described previously. Different jars were opened at each time point and 25 g of Brazil nuts were weighed in porcelain mortars, which were placed in an incubator at 105 °C for 24 h.^19^ Then, the samples were cooled to room temperature in a desiccator and weighed again. The weight loss was calculated as the moisture content of Brazil nuts. Tests were performed in triplicates.

### Sensory analysis of Brazil nuts treated with AITC

Sensory analysis was carried out by a non‐trained panel of 52 volunteers to check if the AITC treatments used in this study would alter the sensory properties of Brazil nuts in comparison with the control. The test performed based on the difference from the control is also known as multiple comparison test. The application of this test aims to demonstrate whether there is a significant difference (*P* < 0.05) between various treatments (samples) and a control reference, estimating the degree of this difference by statistical analysis using ANOVA followed by the Dunnett test. Many evaluators were not necessary for this test because only assertive sensory alterations were questioned and this was not used for launching a new product and/or to check the outcome of major changes in a formulation. Each evaluator was provided with four random coded samples (0.5, 1.0, 2.5 µL L^−1^ AITC and control) and an uncoded control sample that was used by the evaluator as a reference for comparison. Along with the samples the evaluator received water and salt crackers to remove the residual taste between each sample. A score sheet was also provided for each evaluator containing guidelines to perform the test and a hedonic scale of 9 points (1 = extremely better than the control; 9 = extremely worse than the control) to make his/her consideration about each sample.^20^

### Statistical analysis

Graphpad Prism version 6.0 (Graphpad Software Inc., La Jolla, CA, USA) was used for the statistical analysis of data. All experiments were performed in triplicate and differences between groups analyzed with one‐way ANOVA followed by the Tukey post‐hoc test for multiple comparisons. The level of significance considered was *P* ≤ 0.05. The sensory analysis results were also submitted to ANOVA, but the Dunnet test was applied to check the difference between the control and each treatment. Level of significance considered was also *P* ≤ 0.05.

## RESULTS AND DISCUSSION

### Shelf life of Brazil nuts treated with AITC

**Figure 2:**
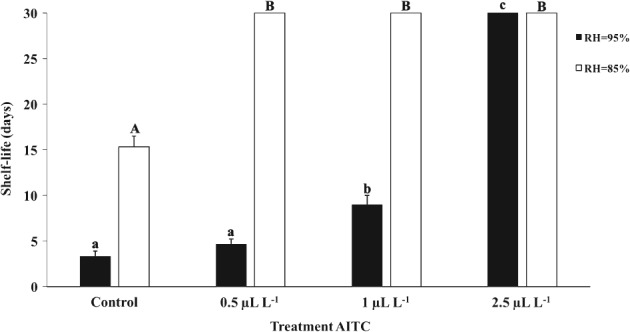
Shelf life (days) of Brazil nuts treated with different concentrations of AITC. Shelf life was determined as the time when mold growth was visually detected. Values are presented as mean ± SD. Different lowercase letters show significant difference (P ≤ 0.05) between treatments at RH = 95%, whereas different uppercase letters denote significant difference (P ≤ 0.05) within treatments at RH = 85%.

Samples were kept under observation for 30 days and the first sign of visual fungal growth was considered the end of their shelf life (Fig. 2). Mold growth was visually detected in the control group after 3 days, 5 days with the application of 0.5 µL L^−1^ AITC and 9 days with 1.0 µL L^−1^ AITC at RH = 95%. No fungal growth was observed in Brazil nuts treated with 2.5 µL L^−1^ of AITC during the 30 days of observation. When the RH was maintained at 85%, the control group was the only one that showed signs of fungal growth, which were firstly noted after 16 days. No growth was observed with the use of AITC at ≥0.5 µL L^−1^. These results demonstrate the problem with uncontrolled moisture in foods and the importance of combining several barriers to avoid microbial growth. The presence of *Aspergillus* species in Brazil nuts is very common,^21^ and its growth is supported by the damp and hot weather of the Amazon region. Then, a longer shelf life of Brazil nuts can be ensured by lowering and controlling the environmental moisture during storage and transportation. Moreover, antifungals, such as AITC, could be used more efficiently when moisture is kept at a lower range. Banerjee *et al*.^22^ evaluated the efficacy of AITC in extending the shelf life of minimally processed shredded cabbage using a cut‐off limit of 10^7^ CFU g^−1^, which is the acceptable limit for fresh cut vegetables and fruits. They demonstrated that microbial count remains below 10^7^ CFU g^−1^ for more than 12 days in samples treated with 0.05 µL mL^−1^ and 0.1 µL mL^−1^ of AITC at 10 °C, while the microbial population was above the limit within 8 days for the control group. However, considering aroma and visual quality, 0.05 µL mL^−1^ of AITC was found to be the optimum concentration to be used for shredded cabbage. Gaseous AITC has also been used to avoid mold growth in bakery products^13^, ^23^ and germination of *Aspergillus flavus* isolated from pistachio nuts at levels ≤5 µL mL^−1^.^24^

### Microbiological analysis and aflatoxins determination

**Figure 3:**
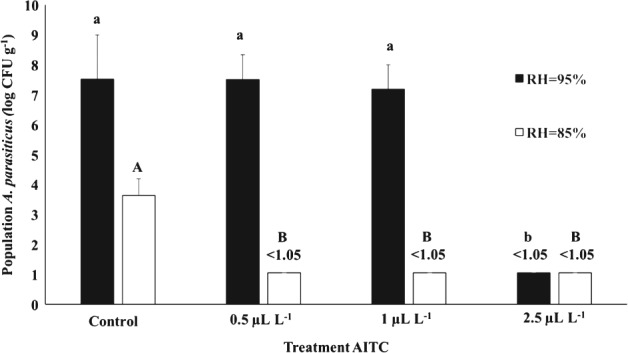
Population of Aspergillus parasiticus CECT 2681 in Brazil nuts treated with different levels of AITC at RH = 95% (black) and RH = 85% (white). The detection limit of the experiment was 1.05 log CFU g^−1^. Values are presented as mean ± SD. Different lowercase letters show significant difference (P ≤ 0.05) between treatments at RH = 95%, whereas different uppercase letters denote significant difference (P ≤ 0.05) within treatments at RH = 85%.

Comparing control groups of both humidity levels tested, *A. parasiticus* grew faster at RH = 95%, reaching a population of 7.5 log CFU g^−1^ after 30 days (Fig. 3). Moreover, the fungus produced 1860, 210 and 370 µg kg^−1^ of AFB_1_, B_2_ and G_2_, respectively (Table 2). On the other hand, *A. parasiticus* population was 3.6 log CFU g^−1^ after 30 days at RH = 85%, which was similar to the initial inoculum. Although the fungal population was not altered during 30 days, AFs were produced at 2.84, 4.95 and 5.57 µg kg^−1^ for AFB_1_, B_2_ and G_2_, respectively. Therefore, humidity represented an important barrier for fungal growth since 10% reduction was implicated in a 3.9 log CFU g^−1^ difference in *A. parasiticus* population. Moreover, AFs production was drastically reduced at a lower RH. No fungal growth was observed in Brazil nuts treated with 2.5 µL L^−1^ of AITC in both 85% and 95% RH and, consequently, no AFs were detected at this level. The lowest level of AITC (0.5 µL L^−1^) applied in this study was not able to inhibit mold growth or AFs production with RH = 95%, but this same treatment reduced *A. parastiticus* and AFs to undetectable levels at RH = 85%. This result again emphasizes the importance of using hurdle technology, in which 10% reduction in RH resulted in greater efficiency of AITC, even at a very low dose.

**Table 2:** Concentration of AFs present in Brazil nuts contaminated with Aspergillus parasiticus CECT 2681 and treated with 0.5, 1.0 and 2.5 µL L^−1^ of AITC at RH = 95% and RH = 85%

Mycotoxin (µg kg^−1^)	AITC concentration (µL L^−1^)			
	Control	0.5	1.0	2.5
	RH 95%			
AFB_1_	1860 ± 190^a^	2210 ± 120ª	920 ± 10^b^	<LOQ^c*^
AFB_2_	210 ± 10^a^	270 ± 60^a^	120 ± 10^b^	<LOQ^c^
AFG_2_	370 ± 50^a^	380 ± 20^a^	270 ± 10^b^	<LOQ^c^
	RH 85%			
AFB_1_	2.84 ± 0.14^a^	<LOQ^b^	<LOQ^b^	<LOQ^b^
AFB_2_	4.95 ± 0.52^a^	<LOQ^b^	<LOQ^b^	<LOQ^b^
AFG_2_	5.57 ± 1.10^a^	<LOQ^b^	<LOQ^b^	<LOQ^b^

Values are presented as mean ± SD.

Different letters show a significant difference (*P* ≤ 0.05) within the same row.

LOQ, limit of quantification.

Treatment with 1 µL L^−1^ AITC at RH = 95% did not reduce microbial growth, but mycotoxin production was reduced by 47.96%, 42.82% and 27.03% for AFB_1_, AFB_2_ and AFG_2_, respectively. Fungal growth and production of AFs were completely inhibited in all samples treated with AITC (0.5, 1.0 and 2.5 µL L^−1^) at RH = 85%, showing the importance of water availability for the development of *A. parasiticus* in Brazil nuts. Hontanaya *et al*.^14^ used gaseous AITC formed directly from oriental mustard flour to inhibit AFs production in nuts obtaining 100% of reduction of AFB_1_, AFB_2_, AFG_1_ and AFG_2_ with the application of 0.5 g of flour and water. Otoni *et al*.^25^ developed a delivery system based on a sachet containing AITC (∼0.220 nL L^−1^) to act as an antimicrobial agent against *A. flavus* in stored peanuts. This system caused a 1 log reduction in the fungal population after 1 week and 4.81 log reduction after 60 days. Quiles *et al*.^13^ tested active packaging devices containing AITC or oriental mustard flour + water to inhibit the growth of *A. parasiticus* and AFs production in fresh pizza crust during 30 days. Fungal growth was only inhibited by devices that delivered 5 µL L^−1^ and 10 µL L^−1^ of AITC within the package. In addition, a sachet containing 850 mg of oriental mustard flour and 850 µL of water was also able to significantly reduce the population of *A. parasiticus*. AFs production was inhibited by all antimicrobial treatments in a dose‐dependent manner. More importantly, AITC in a filter paper at 10 µL L^−1^, AITC in a sachet at 10 µL L^−1^ and the sachet containing 850 mg of oriental mustard flour +850 µL of water could totally inhibit AFs formation.

### Moisture content in Brazil nuts kept at controlled relative humidity

**Figure 4:**
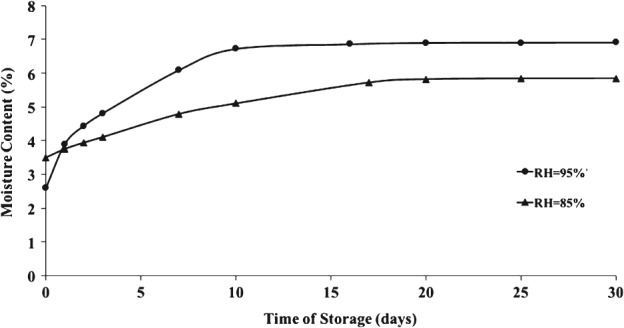
Moisture content in Brazil nuts kept at controlled relative humidity (95% and 85%) after 30 days of storage. Values are presented as mean ± SD.

As expected, Fig. 4 shows that samples kept at RH = 95% acquired more moisture from the environment than those at RH = 85%. This higher moisture content in Brazil nuts led to faster fungal growth as well as larger production of AFs. Correlating moisture content and visual fungal growth, it was observed that *A. parasiticus* did not form macroscopic colonies until nuts reached 4.5–5% of moisture content, which corresponds to ∼30% increment from the initial moisture of 2.5–3.5%. According to Yokoya *et al*.,^26^ Brazil nuts can be stored for up to 8 months in <70% humidity environment with no alterations, but fungi develops rapidly if the same nuts stored at a humidity >80% at temperatures of 27 °C and above, which is very common in the Amazon region. Association of AITC with constant drying of the nuts during bulk storage could prevent the growth of toxigenic and, perhaps, spoilage fungal species.

### Sensory analysis of Brazil nuts treated with AITC

AITC is a very pungent compound that confers distinct flavors to foods. Therefore, its use must be combined to foods that match with spicy flavors or it should be used at levels that do not change the sensory characteristics of a food. Sensory analysis showed no significant difference (*P* < 0.05) between treated Brazil nuts and the control group (Table 3), demonstrating that even the highest dose of AITC (2.5 µL L^−1^) did not alter sensory properties of Brazil nuts. Although AITC has been used to improve food safety and shelf life in several studies, only a few have evaluated the impact of this compound in the sensory properties of the food studied. Moreover, we could not find a single study that examined the effect of AITC in the sensory characteristics of nuts.

**Table 3:** Effect of AITC treatment on the overall acceptability of Brazil nuts using a hedonic scale from 1 (extremely better than the control), 5 (not better or worse than the control) and 9 (extremely worse than the control)

Control	AITC treatment		
	0.5 µL L^−1^	1.0 µL L^−1^	2.5 µL L^−1^
4.63 ± 1.80^a^	4.42 ± 1.46ª	4.62 ± 1.87ª	4.21 ± 2.15ª

Values are presented as mean ± SD.

Different letters show significant difference (*P* ≤ 0.05) within the same row.

Nadarajah *et al*.^27^ evaluated the sensory acceptability of cooked ground beef patties containing different levels of mustard flour used as source of AITC to kill *Escherichia coli* O157:H7 inoculated in the samples. The results of the sensory evaluation showed that there were no significant differences in the acceptability of meat treated with 5% or 10% mustard flour. However, panelists could distinguish untreated controls from mustard treatments, but considered the mustard‐treated meat to be acceptable. The application of gaseous AITC (1.34–6.70 mg L^−1^) on gilthead sea bream (*Sparus aurata*) fillets yielded a distinct but pleasant flavor and contributed to a 8‐day extension of product shelf life.^28^

Chacon *et al*.^29^ evaluated the acceptability to consumers of dry fermented sausages treated with AITC to reduce *E. coli* O157:H7 contamination. Sausages containing 500 µL kg^−1^ AITC were considered acceptable although spicy by panelists. Moreover, gaseous AITC has also been applied in bread to avoid mold growth and the volatile compound was only recognized at concentrations higher than 2.4 µL L^−1^ (gaseous phase) in rye bread and 1.8–3.5 µL L^−1^ in hot dog bread.^23^

## CONCLUSION

Gaseous AITC at ≥0.5 µL L^−1^ combined with RH = 85% could completely inhibit the growth of *A. parasiticus* and AFs production, while at RH = 95% total inhibition was achieved only with 2.5 µL L^−1^. These results confirmed the potential of this compound to be used to preserve foods against fungal spoilage by inhibiting mycotoxin synthesis. Furthermore, it was demonstrated that combination of AITC with hurdle technologies, such as environmental humidity reduction, can further enhance its efficiency, generating economically viable solutions for families that work on the harvest of Brazil nuts. Once harvested, nuts could be stored in bags and fumigated with small doses of AITC to prevent the growth of *Aspergillus parasiticus* and, probably, other fungal species for long periods of time. This could help to avoid aflatoxin contamination in Brazil nuts and, consequently, improve safety standards for its exportation. Finally, sensory characteristics of nuts are not altered even when 2.5 µL L^−1^ AITC was used, and this level could be used as a starting point to evaluate the effect of gaseous AITC to avoid mold growth in Brazil nuts in a pilot scale.
